# Applications of Carbon Nanotubes for Lithium Ion Battery Anodes

**DOI:** 10.3390/ma6031138

**Published:** 2013-03-21

**Authors:** Zhili Xiong, Young Soo Yun, Hyoung-Joon Jin

**Affiliations:** Department of Polymer Science and Engineering, Inha University, Incheon 402-751, Korea; E-Mails: chilebear88@inha.edu (Z.X.); ysyun@inha.edu (Y.S.Y.)

**Keywords:** carbon nanotubes, anode, lithium-ion battery, morphology

## Abstract

Carbon nanotubes (CNTs) have displayed great potential as anode materials for lithium ion batteries (LIBs) due to their unique structural, mechanical, and electrical properties. The measured reversible lithium ion capacities of CNT-based anodes are considerably improved compared to the conventional graphite-based anodes. Additionally, the opened structure and enriched chirality of CNTs can help to improve the capacity and electrical transport in CNT-based LIBs. Therefore, the modification of CNTs and design of CNT structure provide strategies for improving the performance of CNT-based anodes. CNTs could also be assembled into free-standing electrodes without any binder or current collector, which will lead to increased specific energy density for the overall battery design. In this review, we discuss the mechanism of lithium ion intercalation and diffusion in CNTs, and the influence of different structures and morphologies on their performance as anode materials for LIBs.

## 1. Introduction

Traditional energy resources are depleting day after day, and energy storage devices are receiving considerable attention. Lithium ion batteries (LIBs) are representative energy storage devices based on electrochemical energy storage and conversion [1]. With the discovery of highly reversible, low-voltage Li-intercalation carbonaceous materials, Sony realized the commercialization of Li_x_C_6_/Li_1−x_CoO_2_ cells in 1991 [1]. The favorable electrochemical performance of LIBs regarding energy and power densities, as well as the progress in cell design and manufacturing, have made LIBs greatly successful for mobile electronics. Extensive research has been carried out over the past decade, which has led to substantial progress in materials and chemistries to improve the battery technologies for application.

LIBs typically consist of a negative electrode (anode), a positive electrode (cathode), and a conducting electrolyte, and store electrical energy in the two electrodes in the form of Li-intercalation compounds. During charging of the LIBs, lithium ions released from the cathode move through the electrolyte and are inserted into the anode. Upon discharging, lithium ions are extracted from the anode and move back to the cathode. Since electrolytes are not responsible for the conduction of free electrons, electrons that complete the half reaction will move through an extra external wire.

LIBs are noted for their impressively high energy density and high open circuit voltage. Despite superior performance, there are many challenges in the design of LIBs, one of which is improving the capacity and cycle life of the anode materials. Historically, graphite has been commonly used as an anode material for LIBs due to its high electronic (in-plane) conductivity as a consequence of the delocalized π-bonds, and appropriate structure for lithium ion intercalation and diffusion [2]. However, the capacity of LIBs based on graphite as an anode material can be theoretically limited, since lithium ions can only combine with every second carbon hexagon in the graphite sheets. The intercalation of lithium into graphite involves one lithium atom per six carbon atoms, *i.e.*, LiC_6_, leading to a limited specific capacity of 372 mAh/g and an observed capacity of 280–330 mAh/g, depending on the type of graphite used [1]. Carbon nanotubes (CNTs), an allotrope of graphite, have been reported to show much improved lithium capacity compared to graphite, due to their unique structures and properties. CNTs have been reported to display conductivities as high as 10^6^ S·m^−1^ and 10^5^ S·m^−1^ for single-walled carbon nanotubes (SWCNTs) and multi-walled carbon nanotubes (MWCNTs), respectively, and high tensile strength up to 60 GPa [3,4,5]. Recently, many investigations have focused on CNT-based anodes for LIBs with varying success, depending on the treatments employed. This paper provides an overview of the recent research of CNTs in LIB anode applications with respect to structural and morphological factors.

## 2. Mechanism of Lithium Ion Intercalation and Adsorption in CNTs

As a promising candidate for LIB anode materials, CNTs have been widely researched with regard to lithium ion intercalation, adsorption, and diffusion, both theoretically and experimentally. It has been suggested that lithium atoms are stored via two mechanisms: intercalation and alloying [6]. We briefly describe the intercalation and diffusion of Li at different sites on CNTs.

Many studies have been performed in order to investigate the mechanism by which lithium ions are stored in CNTs, including theoretical works [7,8,9,10,11,12,13,14,15,16,17,18,19,20,21,22]. Yang *et al.* [7] proposed a surface mechanism by which the naked surface of CNTs and carbon nanoparticles are able to store lithium species, through investigation of the electrochemical intercalation of lithium into raw end-closed CNTs. Since the CNTs examined were end-caped, and no extra treatments were employed, the lithium adsorbed can only be localized at the surface of CNTs. Zhao *et al.* [8] applied first-principles methods to study the Li intercalation of both zigzag SWCNTs and armchair SWCNTs. They found that both the outside and inside of the nanotubes are susceptible to lithium intercalations, and achieved a high Li density. In order to clarify which sites are more favorable for lithium adsorption, Senani *et al.* [9] investigated the adsorption of lithium ions on the surface of (12,0) SWCNTs by *ab initio* quantum chemical calculations. According to their research, the adsorption of a single lithium ion on the inside of the SWCNTs is favored compared to the outside, since the lithium adsorption energy of one lithium atom on the inside is −0.98 eV, while that on the outside is −0.86 eV. They have also shown that after the lithium attachment, charge is transferred from lithium ions to CNTs, and the bonds between Li atoms and CNTs have ionic properties. They supposed that the amount of charge transfer should be dependent on the radii of curvature of CNTs. It is thus important to investigate this feature of CNTs with different radii. The multiple attachments of lithium ions on the inside of the CNT model were also researched, and four-lithium insertion into one-layer SWCNTs is the most stable.

The interstitial spaces of CNT bundles resulting from van der Waals forces are expected to display a higher ability to intercalate lithium ions, and consequently, higher energy storage capacity. This has been demonstrated by Shimoda *et al.* [10] who reported that the reversible Li storage capacity of the chemically etched CNTs bundles was increased. Song *et al.* [11] investigated the intercalation and diffusion of lithium ions in a CNT bundle by *ab initio* molecular dynamics simulations. They found that lithium ions can penetrate quickly into CNTs, and into the interstitial spaces between neighboring CNTs. In this work, a new approach for lithium intercalation and storage was observed, in that lithium ions could remain between two neighboring CNTs. However, lithium ions that are located between three neighboring CNTs have very strong adsorption potentials, which make it difficult to remove lithium ions from the bundles of nanotubes. This corresponded favorably to the irreversible Li storage capacity reported in etched CNT bundles [10].

As for the diffusion of lithium in the tubes, there exist two directions, the radial direction and the axial direction. Khantha *et al.* [12] studied the interaction and diffusion of lithium ions in (5,5) armchair CNTs using density-functional theory. For a single lithium ion moving in the radial direction, the most favorable positions for Li are along a straight line passing through the center of a six-member carbon ring on one side of the wall, and the midpoint of a C-C bond on the opposite side. After researching the interaction between lithium ions, they found out the interaction energy per Li atom as a function of Li-Li separation clearly indicates an oscillatory character that is especially obvious at larger separations. This can be ascribed to two separate effects, *i.e.*, incomplete screening from the tube and the Li-tube interaction that depends on the position of Li with reference to the hexagonal carbon frame work. Compared to the independent diffusion of individual atoms, the oscillatory ion-ion repulsion favors the concerted diffusion of many Li atoms along the axis. This is consistent with Zhao *et al.’s* [13] results, in which Li displays high mobility along the tube axis with energy barrier less than 47 meV, while the diffusion barrier along the radial direction is as high as 380 meV.

## 3. CNTs (SWCNTs, MWCNTs): Structural Descriptions and Their Application in Anode Materials for LIBs

CNTs have generated great interest among researchers as a unique material since their first discovery by Iijima in 1991 using an arc-charge method [23]. They are considered as one-dimensional structures owing to their high aspect ratio, and SWCNTs and MWCNTs are two of the most important structures. The electrical and thermal conductivities of CNTs are as high as 10^4^ S m^−1^ and 6600 W/mk, respectively [24]. The superior mechanical properties of CNTs, with a Young’s [25] modulus and tensile strength of 1.2 TPa and 50–200 GPa, respectively, make CNTs one of the strongest and stiffest materials. The extraordinary properties and unique structure of both SWCNTs and MWCNTs make them promising materials for a variety of applications [26]. CNTs are especially interesting as one-dimensional hosts for the intercalation of Li, and are regarded as one of the most promising candidates for the anode materials of LIBs.

### 3.1. SWCNTs as Anodes for LIBs

SWCNTs can be envisioned as a rolled-up graphene sheet with diameter ranging from 0.4 to 2 nm, and length up to 1.5 cm [27]. They can be described by a lattice vector (m, n) and a chiral angle, which determine the diameters and chiralities of SWCNTs. Based on the different lattice vectors and chiral angles, CNTs show three chiralities: armchair, zigzag, and chiral. CNTs with (m, m) and (m, 0) types of lattice vectors are termed as “armchair” and “zigzag,” respectively, and are otherwise “chiral”. The chirality of SWCNTs is closely related to the metallic or semiconducting structure of SWCNTs. For an (m, n) nanotube, when the difference between m and n is a multiple of 3, it is metallic; otherwise, the CNT is semiconducting [25,26,28,29,30].

Since the electronic properties of semiconducting and metallic CNTs differ dramatically, it is expected that the electrochemical properties may differ, including the Li storage properties. In LIB devices, electronic conductivity of the anode materials is important especially in granular media in order to bring the electronic carrier as rapidly as possible from the current collector to the electrolyte [2]. Kawasaki *et al.* [31] obtained two kinds of SWCNTs by attempting several methods, and confirmed that the reversible Li ion storage capacity of metallic SWCNTs is about 5 times greater than that of semiconducting ones, through electrochemical charge-discharge measurements. Some theoretical works have been published regarding the preferential interaction of lithium ions with SWCNTs of different chiralities. Udomvech *et al.* [32] concluded that chirality plays a crucial role in Li-tube interactions, and may therefore possibly affect the application of CNTs in LIBs.

Recently, various simple and effective methods for large-scale separation of SWCNTs into populations of single-chirality nanotubes have been reported [33,34]. It is believed that as the technique for single-chirality separation of SWCNTs becomes more and more effective, more reports about the electrochemical performance of SWCNTs with different chiralities in LIB anode applications will be available, and the use of the single-chirality SWCNTs in LIBs will contribute to the development of LIB technology.

### 3.2. MWCNT as Anode for LIBs

MWCNTs, with concentric graphene layers spaced 0.34 nm apart, display diameters from 10 to 20 nm and lengths of hundreds of microns [26]. SWCNTs can be described conveniently by chirality vectors. In contrast, the prediction of electronic properties of MWCNTs, which contain multiple layers of graphene sheets that may have different chiralities, is more complicated. However, due to the multiple rolled layers, MWCNTs are able to insert Li ions in a way similar to graphite, making them a promising candidate as an anode material for LIBs. Except for the inter-planar spacing of graphitic sheets that allows for the intercalation/deintercalation of lithium ions, the hollow cores of MWCNTs are also available for lithium ion intercalation. Therefore, the unique structures of MWCNTs should result in a higher capacity than that of graphite. Much work has been done to verify the excellent electrochemical performance of MWCNT-based anode materials in LIBs applications [35,36,37,38,39,40,41,42,43,44,45,46,47,48,49,50,51,52,53,54].

Shin *et al.* [35] produced relatively pure MWCNTs at moderate temperatures under atmospheric pressure without any preformed substrates. The extremely pure structural characteristic of the MWCNTs produced led to excellent reversibility and small hysteretic loss of the MWCNTs, though with a moderate chemical diffusion coefficient of lithium.

The exfoliation of MWCNTs, which involves peeling off graphene layers during electrochemical insertion of lithium ions, is a unique phenomenon that happens to MWCNTs due to their unique structure. This process increases the anode surface area and thus the formation of a solid-electrolyte interface (SEI) layer, which leads to an increased irreversible capacity of the anode materials. Zhang *et al.* [36] found that this exfoliation process depends on the inner diameter of the tubes and the wall thickness, as well as the size and concentration of the solvated Li^+^ ions. MWCNTs with smaller diameters can be peeled off, resulting in a large irreversible capacity loss in the first cycle. To prevent the exfoliation of graphitic layers, larger CNTs with thicker walls are needed.

Novel structure designs based on MWCNTs with superior electrochemical performance have also been reported. Kang *et al.* [37] developed a 3-dimensional (3D) anode for LIBs. With higher average solid loading of MWCNTs, the 3D anode shows better electrochemical performance, including higher specific capacity, and better cycle stability and C-rate efficiency, compared to a 2D anode with MWCNTs loaded on 2D Cu foil.

Welna *et al.* [38] studied the electrochemical properties of vertically aligned MWCNTs and that of non-aligned MWCNTs for comparison. The structure of the aligned MWCNTs provides more sites at which lithium can be adsorbed, and the intimate contact of the MWCNTs in the aligned structure helps to maintain electrical continuity to the current collector. Both of these help to improve the electrochemical performance of an MWCNT-based anode as a high lithium storage capacity of 980 mAh/g in the first cycle was obtained and stabilized at about 750 mAh/g after more than ten cycles, while for the non-aligned MWCNTs, the values reduced to 158 and 58 mAh/g, respectively. The vertically aligned MWCNTs also displayed substantially greater rate capability than the non-aligned ones, which emphasizes the important role that structure order plays in electrode performance.

## 4. CNTs of Different Morphologies as Anode for LIBS

It is well known that the morphology of CNTs is of great importance for the electrochemical performance of LIBs when CNTs are used as anode materials. This means that the defects, lengths, and diameters of CNTs can influence the performance of CNT-based anode materials. Generally, there are two widely applied methods to modify the morphologies of CNTs: chemical etching and ball-milling. These treatments are reported to result in structural changes and the formation of surface functional groups on CNTs. The structural changes, including the lateral defects on the surface of CNTs and the shortening of the length of CNTs, can increase the Li insertion capacity. The diameter of the CNTs can be controlled by adjusting the synthesizing conditions such as temperature, catalyst, pressure, and so on.

### 4.1. Defective CNTs as Anode Materials for LIBs.

Defective CNTs were reported to be more effective for the adsorption and diffusion of lithium ions [20,54,55,56]. Anodes fabricated from pristine CNTs have been proven to suffer from problems with low practical capacities and high irreversible charge loss due to the formation of a solid electrolyte interface (SEI) and other side reactions [57]. The introduction of defects with a proper method is a promising strategy to overcome the drawbacks of pristine CNTs. For this purpose, chemical etching and mechanical ball milling are commonly applied methods.

**Figure 1:**
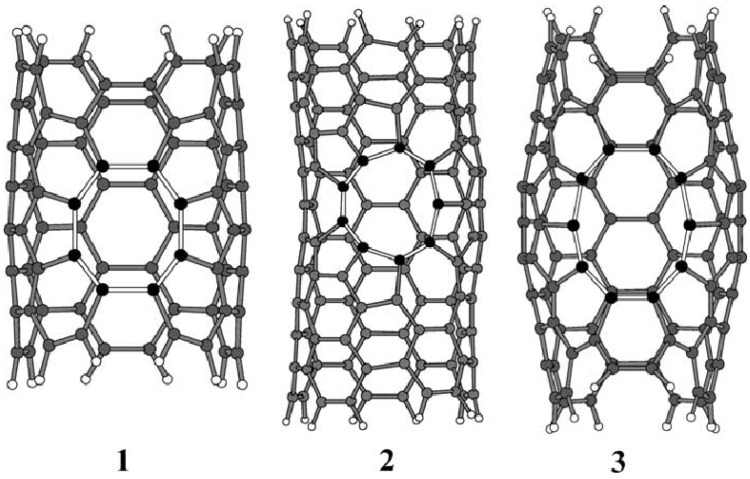
HF/4-31G Optimized structures of nanotubes with 8, 9, and 10-membered ring defects. (Reprinted with permission from [19]. Copyright (2003) by the Elsevier).

CNT defects include holes in the sidewalls, the removal of caps, and fragmentations located at the edges. The presence of holes on the surfaces of CNTs ensures better intercalation and diffusion of lithium ions into the tubes, thereby increasing their capacity. Nishidate *et al.* [20] have investigated the energetics of lithium ion adsorption on defective SWCNTs. The defect formation energy of SWCNTs increases, while the energy barrier for the lithium diffusion lowers as the defective ring becomes large. The results showed that even though lithium had difficulty diffusing into pristine CNTs, as well as n =7 and n = 8 defected CNTs, it was able to diffuse into n = 9 defected CNTs readily. Similarly, by means of *ab initio* calculations, molecular interaction potential (MIP), and MIP with polarization (MIPp) methodologies, Garau *et al.* [19] studied the influence of defects on the diffusion of lithium. Figure 1 shows different optimized structures of three CNTs, from which the effects of defects on the morphology of CNTs can be observed. The MIP energy map and MIPp energy map was used to help to study which defects facilitate the diffusion of lithium ions. The results indicate that ions can enter nanotubes through topological defects (Figure 1) with10-membered rings. The energy barrier for the diffusion through the 9-membered ring is just 9.69 kcal/mol. These results seem to agree with the conclusion by Nishidate *et al.* [20] that larger holes of defects are more favorable for the diffusion of lithium ions.

As discussed, defective CNTs facilitate the insertion of Li ions into the tube and enhance the reversible lithium capacity, which has been confirmed by numerous experimental studies. Eom *et al.* [54] studied the effects of chemical processing on the Li insertion into multi-walled carbon nanotubes. After synthesis on supported catalysts by the thermal chemical vapor deposition (CVD) method, MWCNTs were purified and chemically etched for 5–20 hours [54]. The structural changes of the etched CNTs can be recognized from the Transmission electron microscope (TEM) image. After chemical etching in nitric acid and sulfuric acid for 5, 10, and 20 hours, the lengths of MWCNTs were reduced, and numerous defects were created on their surfaces. Compared with merely purified MWCNTs, the reversible capacity of the etched MWCNTs is considerably increased, from 351 mAh/g (Li_0.9_C_6_) to 681mAh/g (Li_1.8_C_6_). The irreversible capacitance of the etched MWCNTs was also increased with the increase of the Bruinauer-Emmett-Teller (BET) specific surface area, on which an SEI is formed.

**Figure 2:**
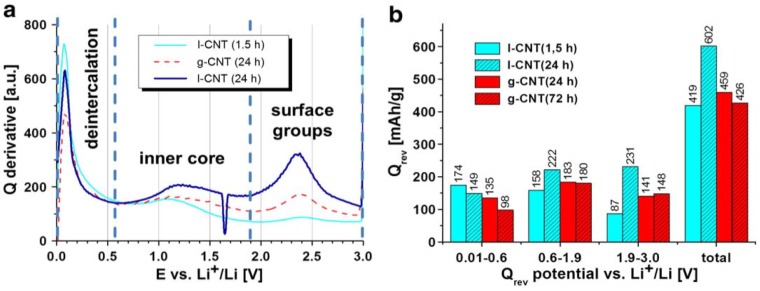
(**a**) Derivative of 1st cycle reversible capacity; and (**b**) reversible capacity of g-CNT (carbon nanotube) and l-CNT, divided into three potential areas of graphene layer deintercalation (0.01–0.6 V), inner core deinsertion (0.6–1.9 V) and extraction from functional groups (1.9–3.0 V); ~0.5 mg/cm^2^, 1 M LiPF_6_ in 1:1 EC: DEC, 50 mAh/g. (Reprinted with permission from [57]. Copyright (2012) by Elsevier).

In another report, Klink *et al.* developed a novel morphology modification method for the gas-phase oxidation of CNTs, which introduces more surface functional groups to CNTs compared to liquid oxidization techniques [57]. In their work, MWCNTs were oxidized by either liquid or gas-phase nitric acid for different durations. In the electrochemical characterizations shown in Figure 2, gas-phase oxidized CNTs (g-CNT) show a much lower amount of initial charge loss (172 mAh/g) than liquid-phase oxidized CNTs (l-CNT) (283 mAh/g) originating from the less-pronounced exfoliation, which was probably caused by an increase of surface carbonyl groups. Therefore, it is supposed that the gas-phase oxidized CNTs are more suitable for battery-related applications, since they show a lower specific initial charge loss as well as easy processing and less mechanical degradation.

In addition to chemical etching with a strong acid, novel chemical methods can be developed to create holes in the walls of CNTs. Oktaviano *et al.* [53] created holes in MWCNTs using CoO_x_ as an oxidation catalyst. The production of defects in CNTs was confirmed by the increased I_D_/I_G_ ratio compared with the pristine MWCNTs observed with Raman spectroscopy, together with the increased specific surface area of the nano-drilled MWCNTs (DMWCNTs). In the TEM images of both pristine CNTs and DMWCNTs, the existence of holes on the basal plane of CNTs can be clearly observed. Electrochemical characterization was performed using the DMWCNTs, purified CNTs, and pristine CNTs as LIB anodes. The DMWCNT-based anode showed the highest specific discharge capacity of 625 mAh/g at a current density of 25 mAh/g after 20 cycles, while capacities of the pristine and purified MWCNTs were 267 and 421 mAh/g, respectively. The relatively higher capacity of DMWCNTs was considered to be a result of the increased lithium storage sites obtained by opening holes in the sidewalls of the MWCNTs.

Except for the CNTs with holes on the sidewall, end-opened CNTs can also show improved ability for lithium ion intercalation and diffusion. Yang *et al.* [55] examined the electrochemical intercalation of lithium into closed and opened CNTs separately. The caps of the CNTs were removed in a silica tube between silica wool plugs, and heated under a flow of air at 700 °C. The discharge capacity of the cap-opened CNTs was 450 mAh/g, which was larger than that of closed tubes. This is considered to be a result of the lithium intercalation into the inner core of opened CNTs, which cannot happen with the closed CNTs, the ends of which were capped by graphitic layers. However, the opened CNTs showed poorer cycling performance than the closed CNTs, which was possibly due to the smaller Warburg prefactor value of the closed CNTs.

### 4.2. CNTs of Different Diameters as Anode Materials for LIBs

The diameter is another parameter of the structure of CNTs that can affect the lithium adsorption and diffusion, both inside and outside the CNTs [13,14,18,58,59]. As mentioned above, lithium ions intercalated into CNTs will interact with CNTs, and the charge transfer between CNTs and lithium ions will further enhance these interactions [9]. The interaction is related to the curvature of the tubes, and will result in different lithium capacities in CNTs [18]. As for MWCNTs, the diameter is also considered to influence the exfoliation of graphene sheets rolled up to form the multi-layer structure of MWCNTs, and will consequently influence the lithium capacities as well [36].

**Figure 3:**
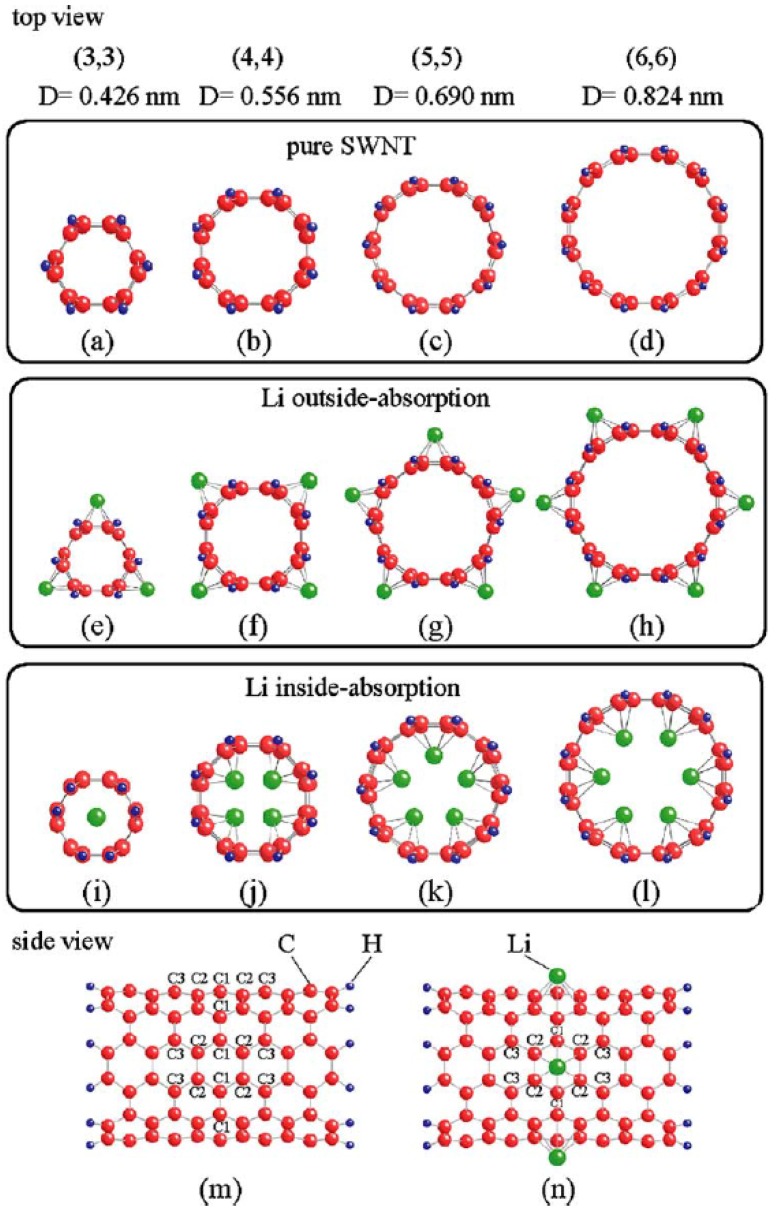
(**a**) C_66_H_12_; (**b**) C_88_H_16_; (**c**) C1_10_H_20_; and (**d**) C_132_H_24_ cluster models used for the calculations of the pure (3,3), (4,4), (5,5) and (6,6) SWNTs; (**e**) Li_3_C_66_H_12_; (**f**) Li_4_C_88_H_16_; (**g**) Li_5_C_110_H_20_; and (**h**) Li_6_C_132_H_24_ cluster models used for the calculations of the Li outside absorption; (**i**) LiC_66_H_12_; (**j**) Li_4_C_88_H_16_; (**k**) Li_5_C_110_H_20_; and (**l**) Li_6_C_132_H_24_ cluster models used for the calculations of the Li inside absorption. The figures (a–l) are top views from the mouth of nanotubes. The figures m and n are side views of the pure and Li outside-absorbed (6, 6) nanotubes. Three kinds of carbon atoms, denoted by C_1_, C_2_ and C_3_, are given in figures. (Reprinted with permission from [14]. Copyright (2004) by Elsevier).

Liu *et al.* [14] conducted a first-principles study on lithium absorption in carbon nanotubes. Their Li-absorbed cluster models are shown in the Figure 3, with limited numbers of lithium atoms absorbed on both the inside and the outside of the CNTs. The Li absorption energy and binding energy of CNTs are dependent on the CNT diameters. The first-principles total energy calculations of Li absorption into CNTs with various diameters showed that with an increase in tube diameter, the external Li adsorption energy decreases, but the internal Li adsorption energy increases. In addition, when the tube diameter is small, the Li adsorption energy for external lithium absorption is larger than that of internal lithium absorption. When the tube diameter is more than 0.824 nm, the external Li absorption energy is similar to the internal Li absorption energy. As for the binding energy, the values for both the pure and Li-absorbed nanotubes increase with the diameter. A close relationship between Li absorption and the diameter of CNTs was demonstrated.

**Figure 4:**
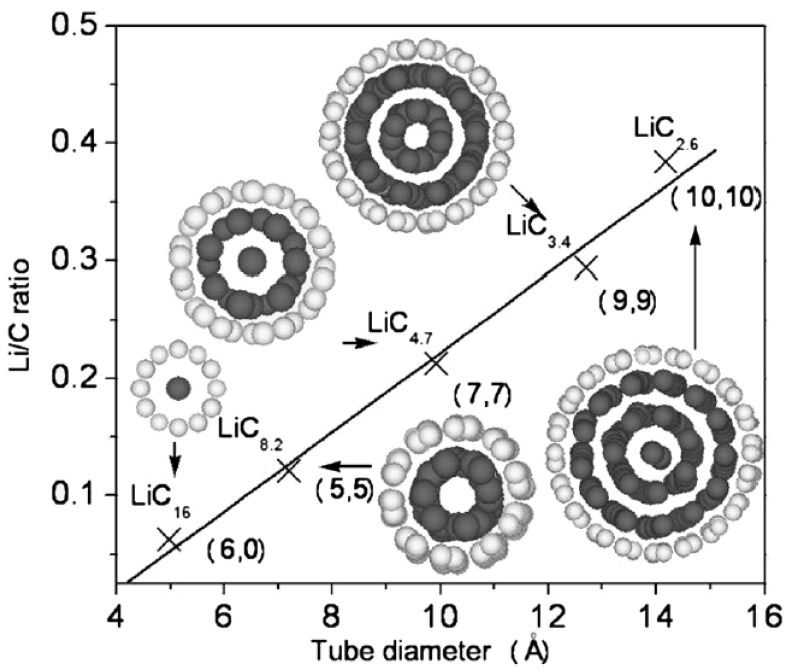
The variation of Li/C ratio as a function of tube diameter. The equilibrium configurations SWNTs filled with Li atoms are shown in the insets of this figure. White and grey balls represent carbon and Li atoms, respectively. (Reprinted with permission from [13]. Copyright (2005) by Elsevier).

More directly, the research performed by Zhao *et al.* [13] showed a clear relationship between the Li/C ratio and the tube diameter. With an increase of the tube diameter, the intercalated lithium atoms tended to form a multi-shell structure (Figure 4) at the equilibrium state, composed of coaxial tubes with a linear chain in the axis, which will improve the lithium capacity. Additionally, Garau *et al.* [18] have shown that the interaction potential at the central region is dependent on the diameter of the nanotubes, and CNTs with a diameter of 4.68 Å have greater interaction energy, thus making them the best candidate for lithium storage as an anode material of LIBs.

**Figure 5:**
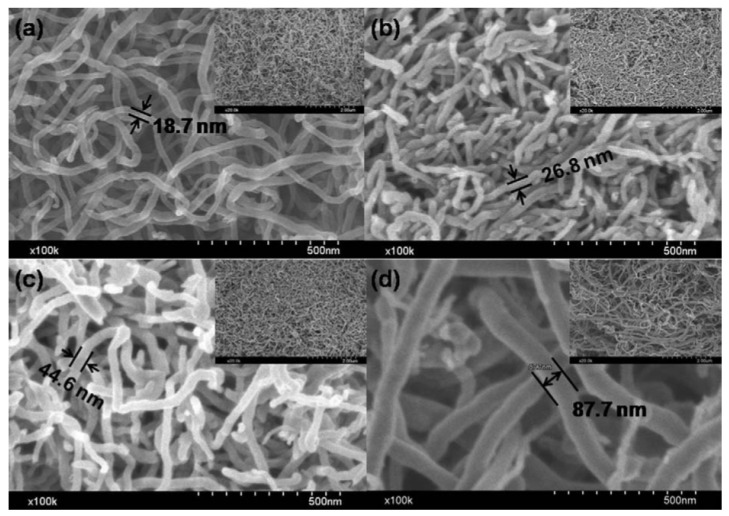
FESEM (Field emission scanning electron microscopy) images of the MWCNTs (multiwalled carbon nanotubes) with diameter range of (**a**) 10–20 nm; (**b**) 20–40 nm; (**c**) 40–60 nm; and (**d**) 60–100 nm. (Reprinted with permission from [58]. Copyright (2012) by Elsevier).

Very recently, Zhang *et al.* [58] performed a study of MWCNTs with different diameters used as anodes for LIBs. Commercial CNTs were confirmed with field emission scanning electron microscopy (FESEM) to display outer diameters of 10–20 nm, 20–40 nm, 40–60 nm, and 60–100 nm (Figure 5). The difference in the diameter range of the MWCNTs was shown to lead to different electrochemical performance when used as anode materials for LIBs. The 50th charge and discharge curves of the MWCNT electrodes showed that electrode fabricated from CNTs with diameters of 40–60 nm displayed better performance, with the highest capacity of 187.4 mAh/g, as well as long cycle life among all the samples, and excellent coulomb efficiency of up to 101.9%.

### 4.3. CNTs of Different Lengths for LIB Anode Materials

Another important influential morphological factor is the length of CNTs, which has also been reported to be able to influence the lithium diffusion of CNTs. The reason for this could be that short CNTs with lateral defects facilitate easier intercalation and deintercalation of Li ions. Lithium ions inserted into CNTs undergo a one-dimensional random walk inside the carbon nanotube, and effective diffusion will decrease if the tube is too long, since the lithium ions are able to enter, but seldom exit [60]. Commonly used chemical etching and ball-milling methods can be used not only to create defects in CNT structures, but also cut CNTs into shorter lengths [61]. However, CNTs produced this way may contain a large amount of surface functional groups, which can lead to substantial voltage hysteresis. Gao *et al.* [62] thought that is can be at least partially related to the kinetics of lithium diffusion in the inner cores of CNTs, and indicated that the hysteresis could be reduced by cutting the nanotubes into shorter segments in their preliminary experiments.

Shorter CNTs can be synthesized directly under proper conditions. Yang *et al.* [63] led a comparative study of the electrochemical properties of short CNTs and long CNTs as anode materials for LIBs. Short CNTs and long CNTs were synthesized by a co-pyrolysis method [64] and the common CVD [65] technique, respectively. The morphologies of short CNTs (CNT-1) and long CNTs (CNT-2) obtained by TEM images which showed that the short CNTs display a length of 150–400 nm, while that of the long CNTs is more than several micrometers. Such large differences in morphology and structure between the two kinds of CNTs have led to different electrochemical performance when used as anode materials for lithium ion batteries. The short CNTs displayed reversible capacities of 266 and 170 mAh/g at current densities of 0.2 and 0.8 mA/cm^2^, respectively, which were almost twice the values obtained with the long CNTs. The kinetics properties have shown that short CNTs display a much lower resistance in a surface film as well as lower charge-transfer resistance than long CNTs. The lithium diffusion coefficients (D_Li_) of both the long and short CNTs decrease as the voltage drops, but for short CNTs, the magnitude of the D_Li_ variation was smaller. Consequently, it was proven that short CNTs are a more promising candidate as an anode material for LIBs compared with longer ones.

**Figure 6:**
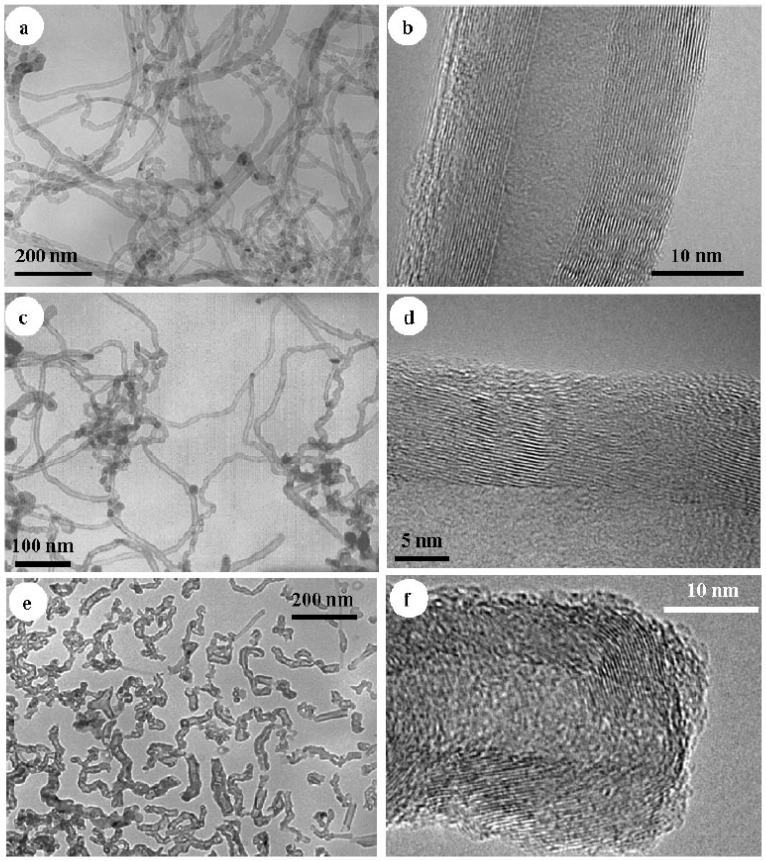
Transmission electron microscopy (TEM) and high-resolution TEM images of (**a,b**) very long; (**c,d**) long; and (**e,f**) short CNTs. (Reprinted with permission from [66]. Copyright (2007) by Wiley).

**Figure 7:**
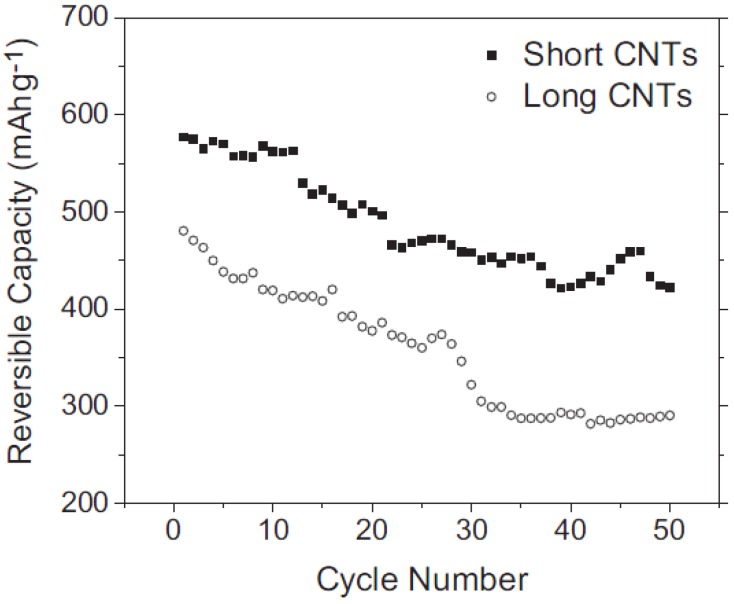
Variation of *C*_rev_ with number of cycles at a current density of 25 mAg^–1^. Note that the capacity becomes stable after 30 cycles. (Reprinted with permission from [66]. Copyright (2007) by Wiley).

A novel solid-state cutting method was developed by Wang *et al.* [66] to prepare short CNTs from conventional micrometer-long entangled CNTs. This solid-state method involves depositing Ni onto CNTs followed by oxidation of Ni at 400 °C. The NiO particles produced then react with CNTs at an even higher temperature of 900 °C, cutting the CNTs into shorter ones. These as-produced short CNTs are about 200 nm in length, as demonstrated by the TEM image in Figure 6. Electrochemical tests were performed on the short CNTs, and long and very long CNTs. As is shown in Figure 7, the reversible capacity was significantly increased, while the irreversible capacity was slightly increased or even decreased as the length of CNTs decreased. Other electrochemical properties of the short CNTs like smaller electrical resistance and Warburg prefactor result in better rate performance at high current densities.

**Figure 8:**
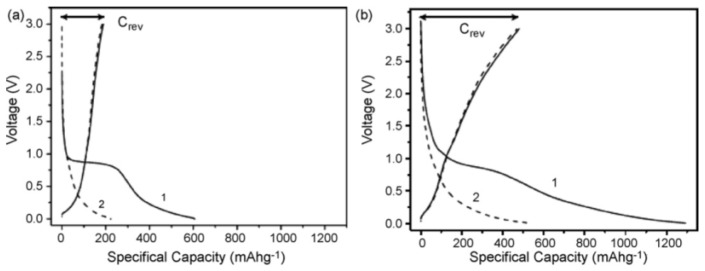

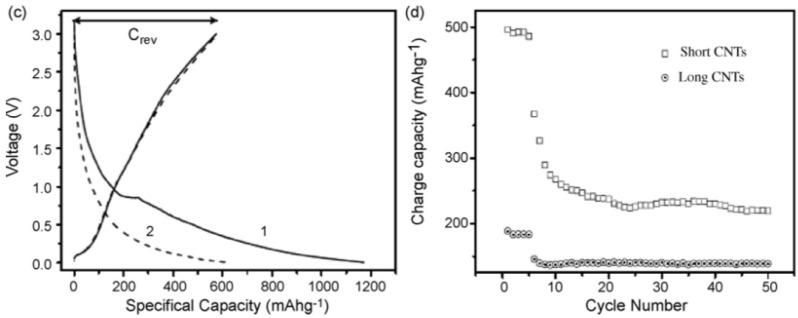
Performances of different CNTs in Li-ion batteries. Discharge/charge curves of Li insertion/extraction into/from conventional (**a**) long; (**b**) short; and (**c**) cut CNTs for the first and second cycles at a current density of 25 mAg^−1^; and (**d**) charge capacities of long and short CNTs as a function of cycle number. (Reprinted with permission from [67]. Copyright (2009) by Elsevier).

In subsequent work, the same group reported for the first time on the use of the Fe compound FeS as a catalyst to produce short CNTs directly [67]. FeS formed during the process promoted the growth of CNTs and limited their length. The directly grown short CNTs were about 200–500 nm in length and 20–30 nm in diameter. These directly produced short CNTs, as well as short CNTs from solid-state cutting and conventional long CNTs, were used in electrochemical tests as anodes in LIB devices. The result showed that the reversible capacity (C_rev_) for the long CNTs was 188 mAh/g, while for the directly grown short CNTs and the cut ones, the values were dramatically increased to 502 and 577 mAh/g, respectively (Figure 8). The stable charge capacities of 230 mAh/g and 142 mAh/g after 20 cycles for short CNTs and long CNTs, respectively, indicate a much better cyclic performance for the short CNTs. The synthetic technique they presented can be used to produce short CNTs in large amounts, and facilitate a wide range of possible application.

### 4.4. Methods of Modifying the Morphology of CNTs

Considering the importance of the morphologies of CNTs in the application as anodes for LIBs, we once again stress the importance of morphology modification methods. Electron irradiation [68,69,70,71] and ion irradiation [72] can be used to produce well-controlled defects on the surfaces of CNTs at a specific location, since beam energy exceeding 80 eV can displace carbon atoms, which can generate crosslinks between individual CNTs. However, there is difficulty using these methods for preparing well-distributed open holes in the bulk of CNTs, since they can only cause point defects on the CNT structure. Chemical etching and ball-milling are two of the most common methods to modify the morphology of CNTs. Both short CNTs and defective CNTs can be produced via either chemical etching or mechanical cutting, or with a combination of these. The chemical etching of CNTs involves the reaction of CNTs with strong acid (nitric acid, sulfuric acid, *et al.*), which produces a great deal of oxygen-containing groups, leading to the destruction of the structure of CNTs in the form of large amounts of defects. Alternatively, ball-milling treatment for an appropriate time could increase both the reversible capacity and coulombic efficiency of CNTs [63]. However, both the chemical etching and mechanical ball-milling methods usually result in the destruction of graphitic structure, inhomogeneous cutting with long CNTs remaining, and a significant loss of raw materials. As mentioned above, new methods have recently emerged, like the direct growth of short CNTs with specific catalysts, and solid-state cutting by depositing NiO or Fe_2_O_3_ particles on CNTs followed by chemical reaction between them at high temperature [66,67]. Compared with conventional chemical etching or ball-milling, these new methods are much more promising for producing short CNTs with narrow length distributions, high dispersion, and low material loss. Additionally, the greater amount of surface functional groups produced using these methods can change the nature of a solid electrolyte interface (SEI), which has an effect on the Li insertion capacity [73]. This is because the surface functional groups can react with the Li ions, forming surface Li carboxylic salt (-COOLi) and/or surface -OLi groups [54]. The SEI formed by Li_2_CO_3_ and EC-, DEC-, DMC-, or PC-based electrolytes can be bonded to the -COOLi or -OLi groups, forming chemically bonded SEI (CB-SEI). This CB-SEI has demonstrated the ability to act as a molecular sieve to reduce the access of solvent molecules to the surface of carbonaceous material, thereby reducing the amounts of the irreversible capacitance in the first insertion process, with an increase in stability of a carbonaceous electrode. We assume that new methods based on new catalysts and proper condition control have potential for producing CNT products with desirable morphologies, and should be researched further.

## 5. Free-Standing CNT “Papers” for LIB Anodes

The investigation and development of flexible power sources has motivated the development of flexible, lightweight, binder-free, and current-collector-free electrodes for LIBs [56,74,75,76,77,78,79,80,81]. In conventional methods for fabricating LIB electrodes, binders are introduced to inhibit the collapse of the active materials from metal current collectors, while current collectors are used to maintain the electrode conductivity [77]. The employment of binders and current collectors contributes nothing but dead weight to the lithium storage, which decreases the energy density of LIBs. Free-standing CNT papers can be fabricated from both SWCNTs and MWCNTs, and then used as anodes for LIBs. Compared with conventional electrode material in the bulky form, free-standing paper electrodes have several advantages [82]. First of all, with the removal of the binders and current collector, the dead weight of an electrode is decreased, leading to the increase of usable capacity and specific energy density for the overall battery design. Secondly, the ease of handling the CNT papers makes them readily shaped into various forms required in a variety of flexible and lightweight electronic devices. 

**Figure 9:**
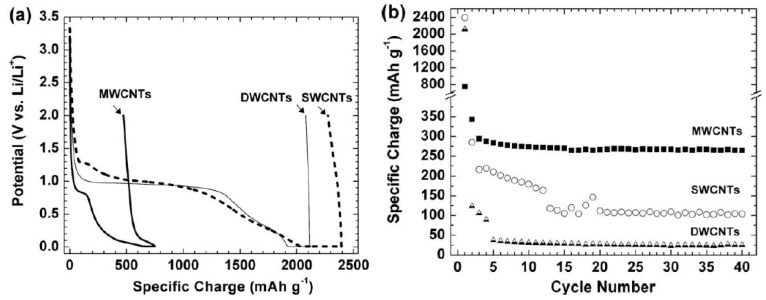
The electrochemical performance of the freestanding CNT films: (**a**) galvanostatic charge–discharge profiles in the 1st cycle; and (**b**) cycling behavior between 0.01 and 2.00 V *vs.* Li/Li^+^ at a specific current of 25 mAg^−1^. (Reprinted with permission from [40]. Copyright (2009) by Elsevier).

The superior performance of free-standing CNT films was confirmed by a series of work. Chew *et al.* [40] conducted a comparative study of the conductive, free-standing, and binder-free CNT films for application as anode materials in LIBs. The flexible free-standing CNT films were made from three different types of CNTs: single-wall, double-wall, and multi-wall CNTs. The preparation of free-standing CNT films involves dispersing 30 mg of the CNTs and 10 wt% of carbon black into 1 wt% of Triton X-100 surfactant in 60 mL of distilled water, followed by a simple filtration process. Figure 9 shows that the MWCNT film exhibits a reversible charge of nearly 300 mAh/g, which is much larger than those of SWCNT film and DWCNT film. The coulombic efficiency of MWCNT film (80% in the 2nd cycle) is also much higher than those of the SWCNT film (59%) and the DWCNT film (57%). Therefore, with advantages of relatively low feedstock price, straightforward vacuum filtration preparation processes, and favorable electrochemical performance, MWCNT films are promising candidates for the anodes of next-generation LIBs.

Li *et al.* [77] developed a novel approach to fabricate a binder-free and current-collector-free nanoporous CNT-film anode on the separator of an LIB. CNTs-separator was obtained via vacuum filtration of CNT dispersion onto an LIB polypropylene separator. CNTs-Cu was prepared by casting CNT slurry onto Cu foil, followed by drying in a vacuum oven at 90 °C overnight. Galvanostatic charge-discharge tests showed that for the first cycle, the specific capacities of the CNTs-separator were 863 and 281 mAh/g, respectively, while for the CNTs-Cu, the values were 768 and 237 mAh/g, respectively. Further characterization showed that the CNTs-separator displayed much better cycling performance and rate performance. The free-standing CNT films, which display superior electrochemical performance, satisfy the requirements of various flexible and lightweight electronic devices.

We speak highly of the “paper-like” electrode due to their special advantages over other types of electrodes, as mentioned above. However, attention should also be paid to overcoming existing challenges, such as the first cycle charge loss and paper crystallinity for free-standing CNT-based anodes [82]. In future developments of paper-like electrodes based on CNTs, CNTs could be combined with conducting polymers [81], or other high-capacity anode materials, like silicon [74] or germanium [79]. The flexibility and improved electrochemical performance of these composite film anodes will surely lead to wider application as anodes for LIBs.

## 6. Conclusion and Future Prospects

In recent years, many efforts have been made to fabricate anodes from raw CNTs, in attempts to replace graphite-based anodes. Varying success has been achieved depending on the synthesis methods and treatment methods applied. However, the highly irreversible capacity of CNT-based anodes together with small improvements in lithium storage capacity and cyclability compared with graphite-based anodes make it difficult to use them for replacing graphite-based anodes in LIB anode applications. It seems that the most promising attempts at improving LIB anodes may come from the combination of CNTs with other lithium storage compounds with elaborate nanostructure designs. The lithium storage compounds employed include metals, transitional metal oxides, and other inorganic materials like silicon [83]. Compared with pure carbon-based anode materials, these lithium storage compounds have much higher lithium storage capacities. However, they suffer from problems with relatively low electronic conductivity or volume changes, which have limited their applications in LIB anodes. Taking these problems into consideration, CNTs are being used as additives to enhance the conductivity of these composites and reduce the structural degradation. Even though great progress has been made in this field, we think that further research should be carried forward for designing new and effective nanostructures using high-capacity lithium storage compounds, and taking advantage of the different morphologies of CNTs.
